# Integrated single-cell RNA-seq analysis identifies immune heterogeneity associated with KRAS/TP53 mutation status and tumor-sideness in colorectal cancers

**DOI:** 10.3389/fimmu.2022.961350

**Published:** 2022-09-12

**Authors:** Xiaoyu Liu, Xu Xu, Zhuozhuo Wu, Qungang Shan, Ziyin Wang, Zhiyuan Wu, Xiaoyi Ding, Wei Huang, Zhongmin Wang

**Affiliations:** ^1^ Department of Radiology, Ruijin Hospital, Shanghai Jiao Tong University School of Medicine, Shanghai, China; ^2^ Department of Pediatrics, Ruijin Hospital, Shanghai Jiao Tong University School of Medicine, Shanghai, China; ^3^ Department of Radiology, Ruijin Hospital Luwan Branch, Shanghai Jiao Tong University School of Medicine, Shanghai, China

**Keywords:** tumor immune microenvironment, colorectal cancer, clinical prognosis, therapeutic targets, tumor sideness, KRAS mutation, TP53 mutation

## Abstract

**Background:**

The main objective of this study was to analyze the effects of KRAS/TP53 mutation status and tumor sideness on the immune microenvironment of colorectal cancer using integrated scRNA-seq data.

**Methods:**

A total of 78 scRNA-seq datasets, comprising 42 treatment-naive colorectal tumors, 13 tumor adjacent tissues and 23 normal mucosa tissues were included. Standardized Seurat procedures were applied to identify cellular components with canonical cell marks. The batch-effect was assessed and corrected using harmony algorithm. The scMetabolism algorithm was used for single-cell metabolic analysis. The results and clinical significance were further validated using immunofluorescent-staining and TCGA-COAD datasets. Immune-infiltration scores of bulk-RNA-seq data were estimated using ssGSEA. The presto-wilcoxauc algorithm was used to identify differentially enriched genes or pathways across different subgroups. Two-sided p-value less than 0.05 was considered statistically significant.

**Results:**

We refined the landscape of functional immune cell subtypes, especially T cells and myeloid cells, across normal mucosa, tumor adjacent and tumor tissue. The existence and function of two states of exhausted CD8^+^ T (Tex) subtypes in colorectal cancer, and FOLR2^+^ LYVE1^+^ macrophages indicating unfavorable prognosis in colorectal cancer were identified and validated. The diverse tumor mutation status reshaped the immune cell function and immune checkpoint ligands/receptors (ICLs/ICRs) expression pattern. Importantly, the KRAS/TP53 dual mutations significantly reduced the major energy metabolic functions in immune cells, and promoted the cell-to-cell communications towards immunosuppression in colorectal cancers. The results revealed LAG3, CD24-SIGLEC10 and HBEGF-CD9 pathways as potential therapeutic targets for dual mutant colorectal cancers.

**Conclusions:**

We revealed that the immune microenvironment underwent a gradual remodeling with an enrichment of immunosuppressive myeloid cells from normal mucosa to tumor regions in colorectal cancers. Moreover, we revealed the metabolic heterogeneity of tumor-infiltrating immune cells and suggested that the KRAS/TP53 dual mutation may impair antitumor immunity by reducing T and myeloid cell energy metabolism and reshaping cellular interactions toward immunosuppression.

## Introduction

Colorectal cancer is one of the most common gastrointestinal malignancies and the fourth leading cause of cancer-related death worldwide. The 5-year survival rates of colorectal cancer were approximately 65% (1). The primary therapeutic approach for early-stage colorectal cancer is surgical resection, and a combination of chemotherapy and targeted therapy may improve the prognosis of patients with advanced-stage disease (2–4). A subset of patients also responded to immunotherapy that targets PD-1 or CTLA-4 checkpoints (5–8), suggesting the critical role of the tumor immune microenvironment (TME) in the progression and treatment of colorectal cancer. A better understanding of the immunological regulation involved in the colorectal tumorigenesis could help to identify new therapeutic targets and thus improve disease management.

Colorectal cancer and its TME are remarkably heterogeneous, both anatomically and genetically (9–11). The tumor anatomical location, which is commonly described as left- or right-sided colorectal cancers, contributes to the heterogeneity (12, 13). In addition, studies have revealed the genetic heterogenicity of colorectal cancers, as evidenced by concurrent high- and low-frequency mutations and transcription profile drift (10, 14–16). These factors affect the susceptibility of colorectal cancer to pharmaceutical chemicals and prognosis of the patients (12, 17–20). With respect to anatomical heterogeneity, the right-sided intestine originates from embryonic midgut, while the left-sided large intestine originates from embryonic hindgut. This ontogenetic divergency may explain the disparity in gene expression profiles between the left- and right-sided colon, as well as colorectal cancers (13). Clinical data indicated that left-sided colorectal cancer has a better prognosis than right-sided cancers, and showed increased susceptibility to first-line FOLFIRI plus cetuximab therapy (12). Although the underlying mechanisms remain unclear, studies have suggested that the disparity in immune infiltration between left- and right-sided colorectal tumors may partially explain the clinical difference (13, 21–23). Moreover, in-depth analyses are still required to fully elucidate this conceivable biological and immunological distinction.

With respect to genetic heterogeneity, *KRAS* and *TP53* are the most frequently mutant genes in colorectal cancers, with roughly 40% of patients carrying activated *KRAS* mutations. Mutant *KRAS* status has been used as a biomarker of resistance to anti-EGFR agents such as panitumumab or cetuximab in clinical practice (24, 25). It has been demonstrated that over-activated RAS signaling confers selective growth advantages on colorectal cancer cells. Moreover, mutant KRAS may also promote tumor development and immune evasion *via* causing an immunosuppressive microenvironment. The mutant KRAS results in immune evasion by recruiting myeloid-derived suppressor cell (MDSC) and impairing CD8^+^ T cell activity *via* inhibition of IRF2 and activation of CXCL3 production in mouse models with colorectal tumors (26). The over-activated *KRAS* is also associated with the functional attenuation of cytotoxic T cells and antitumor neutrophils in patients with colorectal cancers (27). These results may explain why colorectal cancers with *KRAS* mutations are more likely to gain resistance to immune checkpoint blockade (28, 29). In recent years, single-cell sequencing enables the decoding of cellular complexity and intercellular communication in cancers. In colorectal cancer, studies have delineated comprehensive landscapes of T cells (30, 31), myeloid cells (32), and immune response networks (33), elucidating novel mechanisms for immunotherapies (33–36). Studies have also evaluated immune cell composition and molecular features in primary and liver metastatic colorectal cancers (34, 37). However, few investigations have been undertaken to comprehensively evaluate the effect of tumor sideness or mutation status on the biological and immunological heterogeneity of colorectal cancer.

Here, we integrated 78 colorectal cancer patients with scRNA-seq data to elucidate the effects of tumor sideness and KRAS/TP53 mutation status on the tumor microenvironment. A total of 4 publicly available scRNA-seq datasets, comprising 42 colorectal tumors, 13 tumor adjacent tissues and 23 normal mucosa tissues were finally included. We observed that the immune microenvironment underwent a gradual remodeling with an enrichment of immunosuppressive myeloid cells from normal mucosa to tumor regions. Furthermore, we revealed the metabolic heterogeneity of tumor-infiltrating immune cells and suggested that the KRAS/TP53 dual mutation may impair antitumor immunity by reducing T and myeloid cell energy metabolism and reshaping cellular interactions toward immunosuppression. Finally, we validated the clinical significance of our finding using the TCGA-COAD datasets. We anticipate these findings will offer new light on immune microenvironment of colorectal cancers, as well as aid in the improvement of the therapeutic efficacy based on tumor sideness and *KRAS/TP53* mutation status.

## Methods

### Acquisition of single-cell RNA-sequencing datasets and patient information

First, we searched PubMed for studies on single-cell RNA sequencing of colorectal cancer from January 2017 to January 2022. According to the study design, only scRNA sequencing of whole tumor tissue was included, while studies analyzing flow cytometry isolated T cells or myeloid cells were excluded. To minimum the discrepancies and batch effect across sequencing platforms, only data generated from the 10×Genomics sequencing studies were included. Additionally, studies that provided raw sequencing data (including fastq file or raw reads count matrix) were selected, while studies only providing converted datasets (such as TPM files) were excluded. Finally, 78 samples from 3 studies were included in our study (GSE188711, GSE144735, GSE132257) (**Supplementary Figure 1A**).

### Analysis of scRNA-sequencing data

Generally, the scRNA-seq data were performed according to the standard protocols of Seurat (version 3.0) (38). For each sample, the gene and count features were identified, and cells with less than 200 or more than 6000 features were filtered. Then, cells with mitochondrial RNA percentage > 15 or with ribosome RNA percentage < 3 were further removed. Then, the Doublet Analysis was performed using the DoubletFinder (version 2.0.3) (39) with default settings for each sample, and all potential doublets were removed. Subsequently, the scRNA datasets were merged into a larger Seurat file. The integrated data were normalized, scaled and processed for PCA analysis. The data were further visualized using the t-SNE and UMAP methods, respectively. The *JackStraw* method was used to identify the final PCA components for further analysis. The number of cell clusters were evaluated using the shared nearest-neighbor modularity optimization-based clustering algorithm set a resolution of 0.01, 0.05, 0.1, 0.3, 0.4, 0.5, 0.6, 0.7, 0,8, 0.9, 1.0 gradients, and the threshold of 0.01 was chosen, and all cells were divided into 9 highly distinct subpopulations. Similarly, in the subgroup analysis of T cells and myeloid cells, the resolution value was set as 0.3 and 0.2, respectively. The FindAllMarkers function was used to identified the marker genes of each cluster. To assign cell identities and lineages, differentially expressed genes from each cluster were analyzed to sets of previously reported cell-type markers. These 9 subgroups can be well characterized as nine major cell types in tumor. The harmony software (version 0.1.0) (40) was used to evaluated and remove the batch effect in each subgroup. Subsequently, the cells in each group were reanalyzed according to the standard protocol of Seurat. Differentially expressed genes across different groups were identified using the wilcoxauc function in presto package. The clusterProfiler package (41) was used for KEGG and GO pathway analysis.

### Trajectory analysis

We used Monocle2 (version 2.24.0) (42) and Monocle3 (version 1.0.0) (43) to determine the trajectory of T cells, myeloid cells, and epithelial cells. The data processing and pseudo-temporal ordering were all performed using Monocle 2, and graph-based trajectory inference function from Monocle 3 was used to generate the trajectory tree. Cell clustering and annotation were performed using Seurat (version 4.1.1) according to the standard protocol. The presto package *wilcoxauc* function was used to calculate the differential expressed genes of each cluster. The Clusterprofiler package was used for further GO or KEGG enrichment analysis. The dataset was then imported into Monocle2 for pre-processing, including quality control and selection of highly variable genes. For different cell clusters, the top 500~1000 highly variable genes were used for further analysis. The DDRTree (version 0.1.5) method is used for dimension reduction. Cell trajectory was visualized according to cell subtypes and cell states. The Basic Differential Analysis algorithm is used to identify differently expressed gene according to pseudotime function. BEAM (Branched Expression Analysis Modeling) was used to identify genes that are regulated in a branching-dependent manner. The Seuratwrapper package for Monocle 3 was used to cluster cells and develop learned graphs. In addition, Monocle3 was also used to construct the single-cell trajectories for macrophages. The function *learn_graph* was run with default parameters.

### Analysis of the copy number variation and clonal evolution

We employed the inferCNV (version 1.12.0) software (44) to determine the copy number variation (CNV) in each cell. The inferCNV algorithm is an analysis tool for single-cell copy number variant analysis. The core idea of the algorithm is to compare the genome-scale gene expression intensity of each cell from the experimental group to the average gene expression intensity of reference ‘normal’ cells, and the relative expression intensity of genes on each chromosome is obtained and presented in a heatmap. Massively over-abundant or less-abundant genes can be observed in malignant cells as compared to that of normal cells. The cells of interest and reference cells can be further integrated and analyzed using unsupervised clustering analysis, and the potential malignant in the cells of interest can be identified by the results of unsupervised clustering. First, we extracted the epithelial cell subsets, and used epithelial cells from normal colorectal tissues as reference to determine single-cell CNV of epithelial cells from tumor and adjacent tissues. Next, based on the CNV data, we used unsupervised clustering to classify all epithelial cells into 7 subgroups. We distinguished normal and malignant epithelial cells in tumor and adjacent tissues based on the unsupervised clustering data (groups of cells clustered with normal tissue epithelium without significant copy number amplification or deletion). To verify the results, we next calculated a *CNV_score* of each cell. We found that cell subgroups 4 and 7 had the lowest *CNV_scores*, indicating that they were most likely normal epithelial cells. Then, we generated heatmaps according to the CNV data. A hidden Markov model (HMM) was used to identify potential CNV sites, and the output was denoised using a Bayesian latent mixture model. The phylogenetic tree of tumor evolution was draft using Uphyloplot2 software (45) based on the CNV data, and the phylogenetic tree of each sample was classified according to tumor location and mutation status.

### Identification of crosstalk between immune and non-immune cells in colorectal cancers

The CellphoneDB (46) algorithm and R ‘iTALK’ (version 0.1.0) (47) package were used to evaluate the crosstalk across the immunological and non-immunological cellular components in normal mucosa, tumor adjacent and tumor tissue. The quantification of cell-cell communications by calculating the expression of immune checkpoint genes using ‘iTALK’ has been reported in previous studies (48, 49). The expression level of immune checkpoints is a key factor in the remodeling of tumor immune microenvironment and immunotherapy response, and the quantile or custom-defined values of PD-L1 expression were associated with patient outcomes after immunotherapy in lung cancers and colorectal cancers (50, 51). Thus, in our analysis, the ligand or receptor expression density below the first quartile value was thought to be a false positive connection.

### Analysis of the bulk RNA sequencing data for TCGA-COAD cohort

The Htseq-counts matrix and clinical data of the TCGA-COAD cohort (n=478) were acquired using the TCGAbiolinks package (version 2.20.1). Subsequently, a custom-drafted script was used to transform the Htseq-counts matrix to TPM (Transcripts Per Kilobase of Exon Model per Million mapped reads) data. The canonical gene expression marks of 24 different types of tumor-infiltrating immune cells as well as cell marker identified using scRNA-seq data were set according to a prior publication with minor modifications. Then, the R GSVA package (version 1.40.1) was used for single-sample gene set enrichment analysis. The R ConsensusClusterPlus Package (version 1.58.0) and factoextra package (version 1.0.7) were used for unsupervised clustering analysis.

### Immunofluorescent staining

The standard protocol of the multi-color immunofluorescent staining has been reported in our previous studies. Generally, the colorectal samples were cut into 5mm×5mm pieces, and fixed overnight in 4% PFA. The fixed tissues were embedded in paraffin, and sliced into sequential 8-μm thick slides. Immunofluorescent-staining was performed according to a standard protocol on paraffin-embedded slides. Multicolor immunofluorescence staining was performed using the Tyramide SuperBoost Kits (Invitrogen, Cat#B40912-40926). The Panoramic MIDI was used to scan the IF-stained slides (3DHISTECH Digital Pathology Company, Budapest, Hungary).

### Statistical analysis

All statistical analyses were performed using R software (version 4.1.0) or GraphPad Prism 8.0 (GraphPad software Inc.). The comparison of continuous variables between groups was performed using student t-test or welch t-test. The comparison of continuous variables across multiple groups were was performed using t-test with Bonferroni or Dunnett adjustment. The comparison of enumeration data was performed using chi-square test. The survival curve was drafted using Kaplan-Meier methods, and log-rank test was used to compare the survival difference across different groups. Two-sided p-value less than 0.05 was considered statistically significant.

## Results

### Identification of the cellular components of normal mucosa, tumor-adjacent and tumor tissue

**Figure 1:**
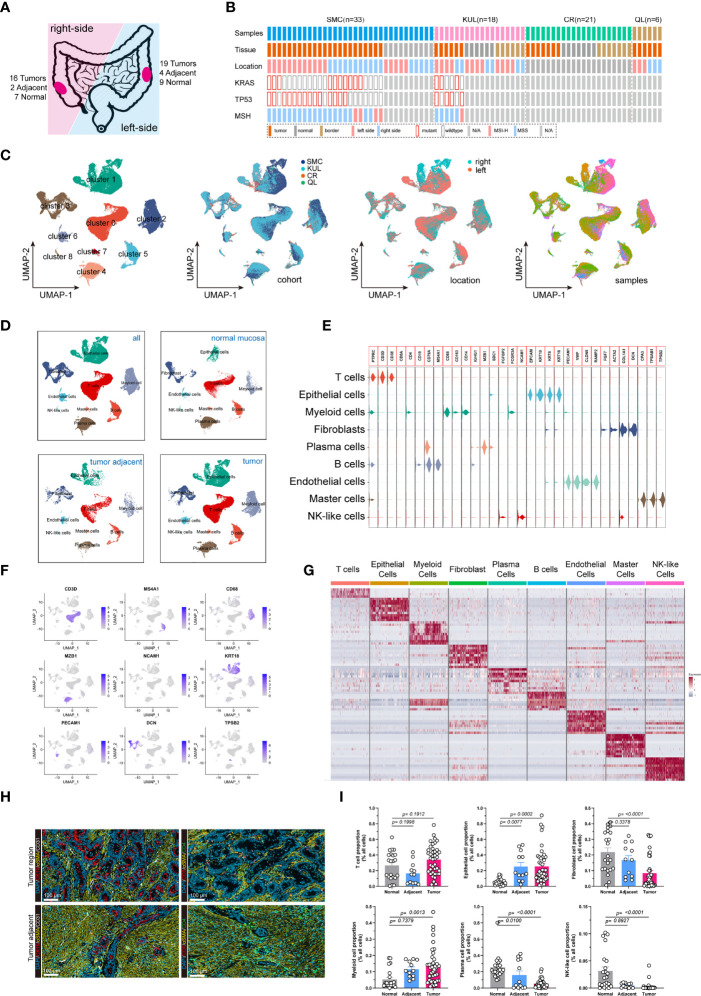
Integrated single-cell transcriptome atlas of colorectal cancer. **(A, B)** Schematic diagram depicted the **(A)** tumor sideness and **(B)** mutations status information. The tumor location indicated the sideness of the colorectal cancer (left-sided or right-sided). The *KRAS* gene primarily included RAS activating mutations such as KRAS^G12D^ and KRAS^G12C^. Whereas the TP53 gene had more diverse mutations sites. The patient cohort information was provided to identify the origins of the patients. **(C)** UMAP dimension reduction of all 71,946 cells, and visualization of the characteristics according to UMAP clusters, cells origin cohorts, tumor location and sample identity. **(D)** UMAP plots displayed 9 major cell-types (epithelial cells, myeloid cells, B cells, T cells, plasma cells, master cells, NK-like cells, endothelial cells and fibroblasts) by tissue types. **(E)** Violin plots displayed the expression of marker genes across the 9 major cell types. **(F)** UMAP plots demonstrated the expression of key marker genes (CD3D, MS4A1, CD68, MZB1, NCAM1, KRT, PECAM1, DCN, TPSB2) across the 9 major cell types. **(G)** Heatmap depicted the top 10 differentially expressed marker genes across the 9 major cell types. For each group, 500 cells were randomly selected to draw the heatmap. **(H)** Representative images of immunofluorescent-stained cytokeratin, α-SMA, CD31, CD4 and CD8 in human colorectal cancer (upper panel) and tumor adjacent tissue (lower panel). **(I)** Bar plot demonstrated the proportion of T cells, Epithelial cells, Fibroblasts, Myeloid cells, Plasma cell, and NK-like cells across normal mucosa, tumor adjacent and colorectal tumor tissues.

We analyzed single-cell RNA (scRNA) sequencing data from 78 treatment-naive samples, including 42 colorectal cancer, 13 tumor-adjacent, and 23 normal mucosal samples (**Figures 1A, B**). The *KRAS* gene primarily included RAS activating mutations such as *KRAS^G12D^* and *KRAS^G12C^*. Whereas the *TP53* gene had more diverse mutations sites. In the subsequent analysis, the tumors were categorized in to 4 groups, including *KRAS* (tumors only bear *KRAS* mutations), *DUAL* (tumors bear *KRAS* and *TP53* dual mutations), *TP53* (tumors only bear *TP53* mutations) and *WT* (neither *KRAS* nor *TP53* are mutated). The patient cohort information was provided to identify the origins of the patients. The detailed clinical information of the samples was presented (**Supplementary Table 1**). After quality control, a total of 132,618 cells with 3,827 median gene count per cell were retained. Then, we categorized all cells into 9 distinct subgroups (**Figure 1C**) through a standard Seurat pipeline. As expected, diverging distribution of cells within each subgroup was observed when depicting the UMAPs based on cohort, tumor location and sample identity (**Figure 1C**). Using previously reported canonical cell markers, the cell subgroups can be identified as T lymphocytes (n= 43,740), Epithelial cells (n= 27,505), Myeloid cells (n=14,680), Fibroblasts cells (n=14,625), Plasma cells (n=13,416), B cells (n=11,971), Endothelial cells (n= 4,082), Master cells (n=1,406), and NK-like cells (n=1,193) (**Supplementary Figure 1B**). The distribution of the cells in normal mucosa, tumor adjacent and tumor tissues were visualized using UMAP plot (**Figure 1D**). Moreover, the cell proportion regarding the sample phenotype was also provided (**Supplementary 1C, D**). The violin plot (**Figure 1E**), feature plot (**Figure 1F**) and heatmap (**Figure 1G**) demonstrated that the expression of canonical marker genes was distinctly expressed across the 9 major cell types. The presence and spatial distribution of epithelial cells, endothelial cells, fibroblasts cells and T cells were also characterized in human colorectal cancers using immunofluorescent staining (**Figure 1H**). Finally, the proportions of cells across each sample were presented. The results showed that the proportions of Epithelial cells and Myeloid cells were increased in tumor tissue, while the proportions of fibroblasts, plasma cells and NK-like cells were decreased in tumor (**Figure 1I**).

### Copy number variation and clonal evolution analysis of malignant cancer cells

**Figure 2:**
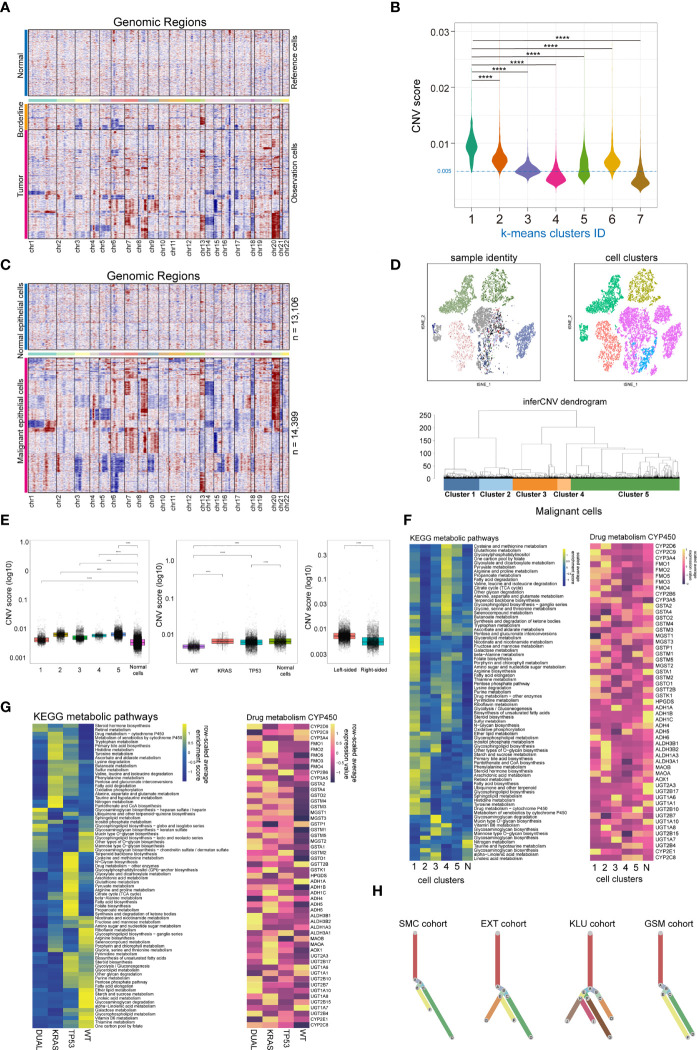
Single cell copy-number variation and clonal evolution of malignant epithelial cells. **(A)** Heatmap depicted copy-number variation (CNV) hierarchical clustering of epithelial cells in colorectal tumor and tumor adjacent tissues, with normal mucosa epithelial cells as a reference. **(B)** Violin plot showed the CNV score across 7 epithelial cell subclusters across normal mucosa, tumor adjacent and tumor tissue, with cells from cluster 4 and 7 classified as normal epithelial cells. **(C)** Heatmap depicted CNV hierarchical clustering of putative malignant epithelial cells (n = 14399). **(D)** The subclusters and distribution patterns of malignant epithelial cells were depicted by the t-SNE (upper) and cluster tree (bottom) plots. **(E)** The boxplots depict the CNV scores of malignant epithelial cells based on subcultures (left), KRAS/TP53 mutation status (middle), and tumor sideness (right). **(F)** A heatmap of significantly altered metabolic pathways (p 0.001) in malignant epithelial cells across multiple subclusters (left), as well as the expression of representative genes in Drug metabolism CYP450 pathways (right). **(G)** A heatmap of significantly altered metabolic pathways (p 0.001) of malignant epithelial cells, as well as the expression of representative genes in Drug metabolism CYP450 pathways (right) based on KRAS/TP53 mutant status. **(H)** Clonality trees depicted the heterogenicity of evolution trajectories across the four cohort. The branches are determined by the proportion of cells in each subclone that contain the respective CNV events. ****p < 0.0001.

Copy number variation (CNV) is a hallmark of malignant cells. Here, we performed CNV analysis to distinguish the malignant cells from the normal epithelial cells, and to evaluate the clonality of colorectal cancers. First, we constructed the CNV atlas of epithelial cells derived from tumor adjacent and tumor tissue with normal mucosa epithelial cells as a reference (**Figure 2A**; **Supplementary Figures 2A, B**). The CNV scores of each epithelial cells (n= 27,505) were calculated, and according to unsupervised cluster analysis, the epithelial cells from tumor and tumor adjacent tissue with minimal CNV and clustered with normal epithelial cells were identified as normal mucosal epithelial cells (n=13,106) (**Figure 2B**; **Supplementary Figure 2C**), while the remainders were identified as malignant epithelial cells (n=14,399). Then, the CNV atlas of all malignant epithelial cells were reconstructed with all normal mucosa epithelial cells as a reference (**Figure 2C**). The malignant cells showed significant copy number amplification on chromosomes 7, 8, 13, 20 and local copy number loss on regions on chromosomes 3, 5, 6, 14 (**Figure 2C**). Unsupervised cluster analysis categorized the malignant cells into 5 clusters (**Figure 2D**). On the t-SNE plot containing malignant cells, there was a substantial overlap between cell clusters and patient identity, indicating that the variation across patients was a main contribution factor for tumor heterogeneity (**Figure 2D**). We also observed tumor cells derived from KRAS/TP53 dual mutant cancers had the highest CNV score (**Figure 2E**, left and middle), while tumor cells from left-sided colorectal cancers had a higher CNV score than right-sided tumors (**Figure 2E**, right). Since metabolic reprogramming plays a key role in colorectal tumorigenesis, we further assessed the metabolic heterogeneity of tumor cells from a single-cell perspective. The metabolic activity of tumor cells was higher than that of normal mucosal epithelial cells, and the metabolic characteristics of the 5 tumor subclusters were significantly different (**Figure 2F**, left). One of the mechanisms leading to this metabolic variability is the distinct expression of genes regulating metabolic pathways (**Figure 2F**, right). The metabolic activity of tumor cells also showed high heterogeneity based on KRAS/TP53 mutation status (**Figure 2G**). Finally, based on the single-cell CNV data, we constructed the clonal trajectory of the colorectal cancers using the UPhyloplot2 algorithm (**Figure 2H**). The results showed that the most samples shared the similar trunk clones. This result is similar to the previous results of bulk sequencing that identified the CNV of colorectal cancer genome with a trunk clone.

### Heterogeneity of the tumor infiltrating lymphocytesin colorectal cancers

**Figure 3:**
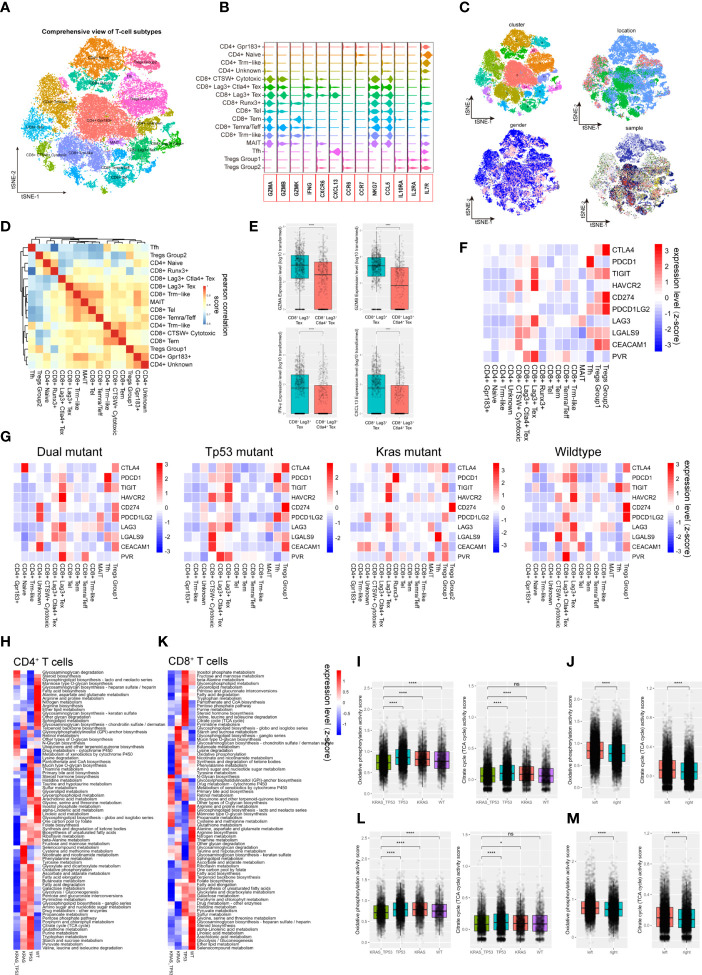
Tumor-infiltrating T cells exhibit molecular and metabolic heterogeneity according to tumor sideness and KRAS/TP53 mutation status. **(A)** The tSNE dimension reduction of all 71,946 T cells (including T cells derived from normal mucosa, tumor adjacent and tumor tissue), and visualization of the characteristics based on tSNE clusters. **(B)** The violin plot displayed the expression levels of key cytokines and receptors across various T cell types. **(C)** Visualization of the T-cell characteristics based on tSNE clusters, cells origin, tumor location and sample identity. **(D)** The heatmap showed spearman correlation between various cell subpopulations based on the top 500 genes with the highest standard deviation. **(E)** Boxplots presented the expression levels of GZMA, GZMB, IFN-γ and CXCL13 in the two Tex (CD8^+^LAG3^+^ Tex and CD8^+^LAG3^+^CTLA4^+^ Tex) groups. **(F, G)** The average expression levels of immune checkpoint molecules across various subtypes of T cells in **(F)** all tumor-infiltrating T cells, and **(G)** the expression according to KRAS/TP53 mutation status. The bar depicted the row-scaled average gene expression level. **(H)** The heatmap of significantly altered metabolic pathways (p < 0.001) of tumor-infiltrating CD4^+^ T cells based on KRAS/TP53 mutation status. The bar depicted the row-scaled pathway enrichment level. **(I, J)** The boxplots showed the levels of Oxidative phosphorylation (left) and Citrate cycle (TCA cycle) activity score of tumor-infiltrating CD4^+^ T cells according to **(I)** KRAS/TP53 mutation status and **(J)** tumor sideness. **(K)** The heatmap of significantly altered metabolic pathways (p < 0.001) of tumor-infiltrating CD8^+^ T cells based on KRAS/TP53 mutation status. The bar represented the row-scaled pathway enrichment level. **(L, M)** The boxplots presented the levels of Oxidative phosphorylation (left) and Citrate cycle (TCA cycle) activity score of tumor-infiltrating CD4^+^ T cells based on **(L)** KRAS/TP53 mutation status and **(M)** tumor sideness. ns, not significant; *p < 0.05; ****p < 0.0001.

T lymphocytes play an important role in cancer immunotherapy. Their variety in cell composition, transcriptomic patterns, and functional features has a profound impact on T cell-based immunotherapy. Despite the fact that the comprehensive T-cell landscape of colorectal cancer has been well characterized, there is a dearth of in-depth analysis demonstrating the impact of tumor phenotype, including tumor sideness and mutation status, on T cell activity. Here, we identified 43,740 lymphocytes from all 78 samples (normal mucosa = 8,459 cells; tumor adjacent = 4,507 cells, tumor = 30,774 cells). After assessment and correction of the batch-effect, we performed PCA analysis and dimension reduction, and categorized the lymphocytes into 16 subgroups, which were majorly recognized as functional CD4^+^ and CD8^+^ T cells based on previously reported T cell markers (**Figures 3A, B**; **Supplementary Figures 3A, B** **)** (31). T cell subgroup characteristics based on tSNE clusters, cell origin, tumor location, and sample identity (**Figure 3C**), as well as the spearman correlation across subgroups (**Figure 3D**), were also presented. Interestingly, the gender group shows a bias phenomenon regarding CD8^+^ Runx3^+^, CD8^+^ CTSW^+^ cytotoxic, and CD8+ exhaustion. Consequently, we investigated the gender-specific gene expression feature of these T cells. We observed that the expression of NKG7, GZMH, GZMA and GZMB in tumor-infiltrating CD8+ CTSW+ cytotoxic cells were significantly increased in female patients, indicating that these T cells have a stronger antitumor cytotoxic effect (**Supplementary Figure 3C**). Notably, two groups of potentially exhausted CD8^+^ T cells (CD8^+^ Tex), LAG3^+^ CD8^+^ Tex and LAG3^+^ CTLA4^+^ CD8^+^ Tex, were identified. The expression of GZMA, GZMB, IFN-γ and CXCL13 were significantly reduced in the LAG3^+^ CTLA4^+^ CD8^+^ Tex (**Figure 3E**), suggesting the terminal state of T-cell exhaustion. Next, we assessed the ICL/ICRs (immune checkpoint ligand/receptors) landscape expressed on T cells. The expression level of clinically targetable immune checkpoint ligand or receptors were quantified in all the T cell subtypes (**Figure 3F**). Notably, tumor sideness had a minimal effect on the ICL/ICRs expression pattern (**Supplementary Figure 3D**), while the ICL/ICRs showed unique distribution patterns based on tumor KRAS/TP53 mutation status (**Figure 3G**). These results suggested that the tumor sideness is not an effective predictor for immune checkpoint blockade-based immunotherapies. For instance, the main contributor of CTLA-4 and TIGHT were Tregs across all groups, the CD8^+^ T subgroups showed decreased expression of PD-1 in KRAS/TP53 dual mutant tumors, and the main contributors of PD-1 in the mutant cancer appeared to be Follicular helper T cells (Tfh cells) (**Figure 3G**).

Next, gene set enrichment analysis (GSEA) of the DGEs was performed according to KRAS/TP53 mutation status, and significantly downregulated metabolic regulating pathways, including Glycolysis, Cholesterol homeostasis, and Myc targets, were observed in CD4^+^ and CD8^+^ T cells from KRAS/TP53 dual mutant cancers (**Supplementary Figures 4A–E**). In addition to the expression levels of cytokines and ICL/ICRs, the immune-metabolic condition of T cells is also tightly associated with antitumor immune function (52). Resting T cells usually have a higher level of oxidative phosphorylation, while T cell activation attenuated mitochondrial respiratory, oxidative phosphorylation and enhanced glucose and glutamine metabolism. We observed a global decrease in CD4^+^ T cell metabolism in KRAS/TP53 dual mutant colorectal cancers (**Figure 3H**). The oxidative phosphorylation and TCA cycle, the two most prominent energy sources for T lymphocytes, in CD4^+^ T cells were both impaired in KRAS/TP53 dual mutant colorectal cancers (**Figure 3I**), indicating the degraded function of these T cells. Interestingly, the CD4^+^ T cells in left-sided colorectal tumors had significantly higher levels of oxidative phosphorylation and the TCA cycle than those in right-sided tumors (**Figure 3J**), suggesting a higher T cell function in left-sided colorectal cancers. This may be one reason why left-sided colon cancer has a better prognosis than right-sided colon cancer in clinical practice. Similar results were also observed in CD8**^+^** T cells (**Figures 3K–M**). Taken together, these results indicated that KRAS/TP53 mutation status had a significant impact on molecular and metabolic function of tumor-infiltrating T cells.

**Figure 4:**
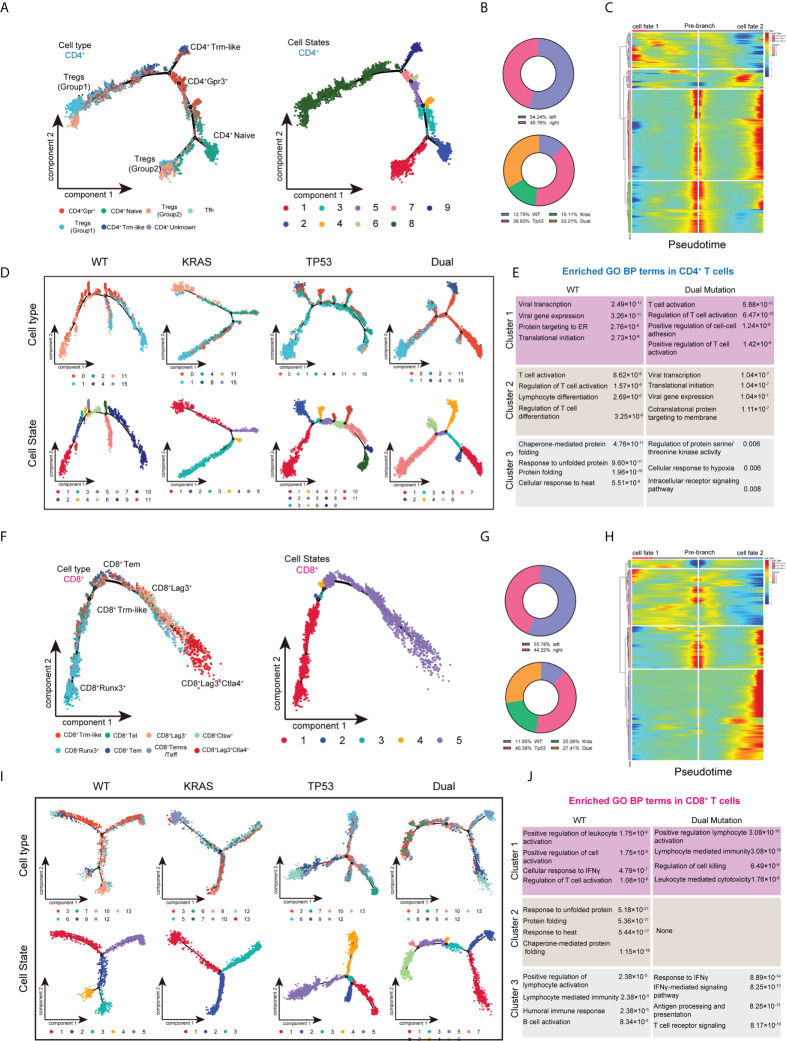
Trajectory analysis of intratumor CD4^+^ and CD8^+^ T lymphocytes cells in colorectal cancers with based on KRAS/TP53 mutation status. **(A)** The trajectory plot (monocle2) showed the dynamics of all tumor-infiltrating CD4^+^ T cell clusters and their pseudotime-associated cell state. **(B)** The proportion of CD4^+^ T cells based on tumor sideness and KRAS/TP53 mutation status. **(C)** According to the BEAM analysis, key genes were hierarchically categorized into four subclusters along the trajectory branching of CD4^+^ T cells. **(D)** The trajectory plot (monocle2) showed that the trajectory path of CD4^+^ T cells were distinct based on tumor KRAS/TP53 mutation status. **(E)** The enriched gene ontology (GO) terms in different BEAM trajectory clusters. **(F)** The trajectory plot (monocle2) showed the dynamics of all tumor-infiltrating CD8^+^ T cell clusters and their pseudotime-associated cell state. **(G)** The proportion of CD8^+^ T cells based on tumor sideness and KRAS/TP53 mutation status. **(H)** According to the BEAM analysis, key genes were hierarchically categorized into four subclusters along the trajectory branching of CD8^+^ T cells. **(I)** The trajectory plot (monocle2) showed that the trajectory path of CD8^+^ T cells were distinct based on tumor KRAS/TP53 mutation status. **(J)** The enriched gene ontology (GO) terms in different BEAM trajectory clusters.

To better understand the effect of mutant KRAS/TP53 on T cell functions. We also analyzed the transcriptome trajectory using Monocle2, which reconstructs putative branching transcriptional trajectories to identify potential relationships across calculated states, to construct the putative branching trajectories based on pseudotime (**Figures 4A–J**). Pseudotime ordering of all CD4^+^ T cells yields a total of 8 cell states organized into 4 main branches (**Figure 4A**). We next analyzed the trajectories of CD4^+^ T cells using branched expression analysis modeling (BEAM) and hierarchical clustering to identify genes enriched across states **(** **Figures 4B, C**; **Supplementary Table 2** **)**. The heatmap clearly showed the enrichment of 64 genes according to pseudotime-based cell states (**Figure 4C**). Additional analysis revealed that the T cell trajectories of KRAS/TP53 mutant tumors were markedly different from those of wild-type tumors, with more trajectory branches (**Figure 4D**). Notably, GO analysis revealed that the expression profiles of a subset of CD4^+^ cells in KRAS/TP53 mutant tumors were enriched for response to hypoxic, implying that they may be affected by the hypoxic microenvironment (**Figure 4E**). While functions of CD8^+^ T cells were impaired in KRAS/TP53 mutant cancers, their cell-state branching trajectory seems to be unaffected by tumor mutation status, suggesting a different phenotypic plasticity compared to CD4^+^ T cells (**Figures 4F-J**).

### The myeloid cells existed different phenotypes with pro- or anti- tumoral functions

**Figure 5:**
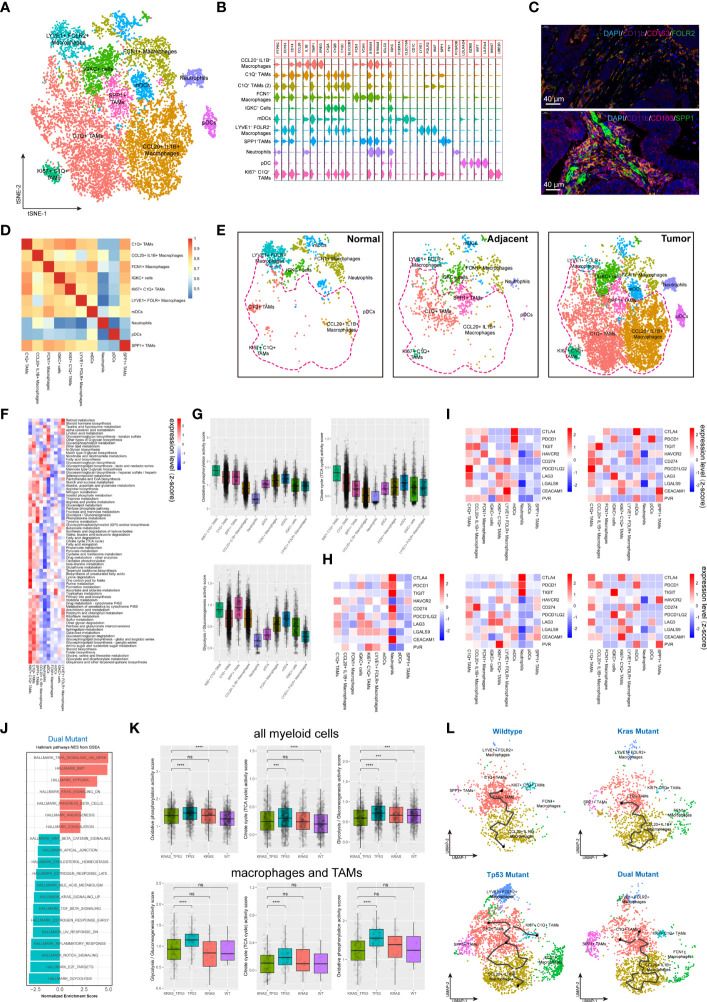
Tumor-infiltrating myeloid cells exhibit molecular plasticity and metabolic heterogeneity according to tumor sideness and KRAS/TP53 mutation status. **(A)** UMAP dimension reduction of 14,680 myeloid cells (containing myeloid cells derived from normal mucosa, tumor adjacent and tumor tissue), and visualization of the features based on UMAP clusters. **(B)** The violin plot presented the expression levels of canonical myeloid cell markers across cell types. **(C)** Representative images of immunofluorescent-stained DAPI, CD11b, CD163, FOLR2 (upper) and SPP1 (lower) in human colorectal cancer. **(D)** The heatmap displayed spearman correlation between various myeloid cell subpopulations based on the top 500 genes with the highest standard deviation. **(E)** Visualization of the distribution of myeloid cell types across normal mucosa (left), tumor adjacent (middle) and tumor tissue (right). **(F)** The heatmap of significantly enriched metabolic pathways (p < 0.001) across tumor-infiltrating myeloid cell types. The bar represented the row-scaled pathway enrichment level. **(G)** The boxplots presented the levels of Oxidative phosphorylation (upper left), Citrate cycle (TCA cycle) (upper right) and Glycolysis/Gluconeogenesis (lower) activity score across distinct tumor-infiltrating myeloid cell types. **(H, I)** The average expression levels of immune checkpoint molecules among different subtypes of **(H)** all tumor-infiltrating myeloid cells, and **(I)** the expression according to KRAS/TP53 mutation status. **(J)** The GSEA analysis of DEGs of myeloid cells derived from KRAS/TP53 dual mutant colorectal cancers. **(K)** The boxplots showed the levels of Oxidative phosphorylation, Citrate cycle (TCA cycle) and Glycolysis/Gluconeogenesis activity score of tumor-infiltrating myeloid cells according to KRAS/TP53 mutation status in all myeloid cells (upper) and tumor-associated macrophages (lower). **(L)** The trajectory plot (monocle3) showed the dynamics of all tumor-infiltrating macrophages subclusters and their pseudotime-associated cell state based on KRAS/TP53 mutation status. ns, not significant; ***p < 0.001; ****p < 0.0001.

Myeloid cells are considered to be the immune cells with the most plasticity. A total of 14,680 myeloid cells were identified in our cohort. The myeloid-derived cells were categorized into 10 subgroups, including macrophages, typical tumor-associated macrophages (TAMs), dendritic cells (DCs) and neutrophils, according to canonical myeloid cell markers (**Figures 5A, B**, **Supplementary Figures 5A, C**) **(** 32). The existence of SPP1^+^ TAMs and FLOR2^+^ TAMs in human colorectal cancers were also validated using immunofluorescent staining (**Figure 5C**). Exception of neutrophils, plasma DCs (pDCs), and SPP1^+^ TAMs, the transcriptome profile of the myeloid cells showed a similarly to some extent (**Figure 5D**). According to the clustering results, C1q^+^ TAMs, SPP1+ TAMs, and CCL20^+^IL1B^+^ macrophages were substantially more abundant in tumor tissue than in non-malignant colorectal tissues (**Figure 5E** **)**. The metabolic features of these 10 subgroups of myeloid cells also differed significantly (**Figure 5F**). The high metabolic activity of Ki67^+^ C1q^+^ TAMs may be related to the characteristics of active proliferation, while the elevated glycolysis activity of SPP1^+^ TAMs is also consistent with the hypoxic tumor microenvironment (**Figure 5G**). The expression of major ICRs (including PD-1, LAG-3, TIGHT and TIM-3) and ICLs (including PD-L1, PD-L2, HAVCR2, LGALS9, CEACAM1, FGL1, NECTIN2, and PVR) were also quantified in the myeloid cell types (**Figure 5H**). The C1q^+^ TAMs were the predominant contributor of immunosuppressive ICLs, including PD-L1, PD-L2, HAVCR2, LGALS9, and CEACAM1. Similarly, to the results found in T cells, the ICL/ICRs expressed on myeloid cells also showed unique distribution patterns based on tumor KRAS/TP53 mutation status (**Figure 5I**). The GSEA analysis of DEGs showed a significant upregulated TNF-α pathway and downregulated inflammatory response pathway in myeloid cells in KRAS/TP53 dual mutant colorectal cancers (**Figure 5J**). Despite the fact that the energy metabolism function of myeloid cells was significantly hindered in KRAS/TP53 mutant cancers, the glycolysis function of TAMs did not appear to be substantially reduced (**Figure 5K**), suggesting adaptation of TAMs to the KRAS/TP53 mutant tumor microenvironment. Finally, trajectory analysis using monocle3 also revealed a more complex evolutionary trajectory of myeloid cells KRAS/TP53 in tumors (**Figure 5L**). In summary, our data indicated that the KRAS/TP53 status significantly affects the cellular states of tumor-infiltrating myeloid cells, particularly macrophages. Since macrophages are important providers of immunosuppressive ligands, patient stratification based on KRAS/TP53 status for individualized treatment may be a viable immunotherapeutic strategy for colorectal cancers.

### Crosstalk between immune and non-immune cells in colorectal cancers

**Figure 6:**
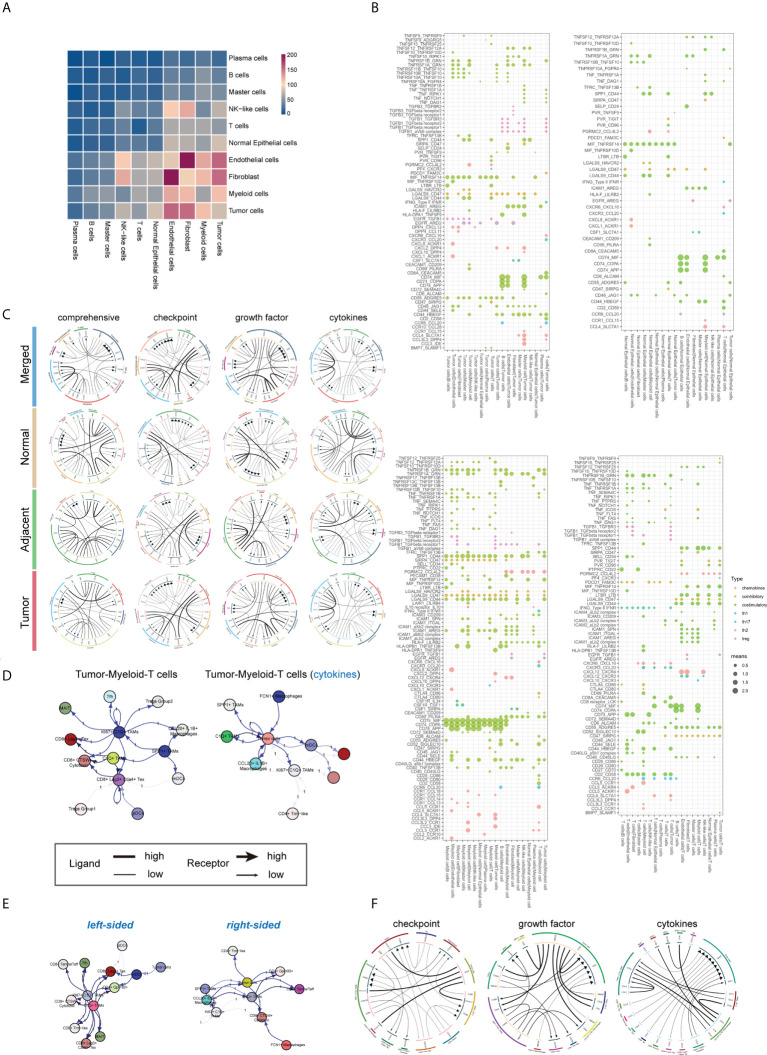
Identification of putative ICR–ICL interactions between cellular subtypes of the tumor microenvironment. **(A)** Heatmap depicted the comprehensive view of putative ICR–ICL connections pairs for each major cell subtypes (including cell derived from normal mucosa, tumor adjacent and tumor tissue). **(B)** CellphoneDB software detected the ligand-receptor connections involving tumor cells (left) and myeloid cells (middle), and T cells (right). The average interaction scores are presented by circle size, while the immune functions involved are indicated by circle color. **(C)** The circular plots depicted the global and functional molecular (immune checkpoints, growth factors, and cytokines) interactions in normal mucosa, tumor adjacent tissue, and tumor tissue. **(D)** The net plot depicted a view (global and cytokines) of the top 20 cell-cell interactions involving tumor cells, **T** cells and macrophages in colorectal cancers. **(E)** The net plot depicted a comprehensive view of the top 20 cell-cell interactions involving tumor cells, T cells and macrophages in left- and right- sided colorectal cancers. **(F)** The circular plots depicted the cell-to-cell interactions across malignant epithelial cells, myeloid cells and T cells in colorectal tumors tissue.

Recent studies have underlined the significance of cell-to-cell communication in the development of diverse malignancies. The CellphoneDB algorithm and R ‘iTALK’ (version 0.1.0) package were used to evaluate the crosstalk across the immunological and non-immunological cellular components in normal mucosa, tumor adjacent and tumor tissue (**Figure 6A**). Compared with normal mucosal epithelial cells, the communication between tumor cells and immune cells was significantly enhanced (**Figure 6B**, left). As expected, myeloid cells and T cells have also showed a strong ability to communicate with the tumor microenvironment (**Figure 6B**, right). Further results revealed complex cell-cell communication networks across cancer and immune cells (**Figure 6C**). Next, we investigated the role of cell-cell communication in TME remodeling in colorectal tissue from the perspectives of immune checkpoints, cytokines and growth factors (**Figure 6D**). We found that the CD274-PDCD1 ligand-receptor pair’s connection was quite weak through the integration analysis. The ligand-receptor communication density identified by the iTALK algorithm is majorly determined by the expression levels of receptor and ligand genes in target cells. In contrast, some inhibitory ICR/ICL pairs such as CD24-SIGLEC10 and LGALS9-HAVCR2 were frequently observed between myeloid cells, master cells, B cells and tumor cells (**Figures 6C, D** **)**. Of note, the immune and non-immune components showed distinct cell-cell communication patterns in normal mucosa, tumor adjacent and colorectal tumors. Tumor tissue presented more unique inhibitory ICR/ICL pairs such as CD80-CTLA4 and CD70-CD27 (**Figure 6D**). Endothelial cells are the primary involvers of cell crosstalk *via* growth factor in tumor adjacent region, while malignant epithelial cells are dominantly involved in the tumor tissues. In tumor tissue, the malignant epithelial cells not only produced growth factors such as HB-EGF, but also expressed CD44 and CD9 receptors to receive growth signaling, further promoting the tumor progression (**Figure 6D**).

Next, we detailed the cell-to-cell interactions across malignant epithelial cells, myeloid cells and T cells in colorectal tumors tissue (**Figures 6E, F** **)**. The data demonstrated that Tregs were the primary source of CTLA4, while Tfh produced the CD40L signal, suggesting that CD4^+^ T cells play a complicated role in tumorigenesis (**Figure 6F**). For tumor cells, CD24 was the most important intrinsic molecular providing a “don’t eat me” signal, meanwhile, SDC4 was the most important receptor for cytokine signaling (**Figure 6F**). Finally, our data showed that tumor sideness had minimal effect on cell-to-cell communication, however KRAS/TP53 mutation status had a significant impact (**Figures 6G, H** **)**. For instance, compared with KRAS/TP53 wildtype colorectal cancer, the CD24-SIGLEC10 immunosuppressive signaling and the HBEGF-CD9 tumor growth signaling across tumor and immune cells were dramatically increased in KRAS/TP53 dual mutant tumors (**Supplementary Table 3**), which was highly consistent with the high malignancy and poor prognosis of KRAS cancers. Taken together, we demonstrated the cell interactions across immune cells and non-immune cells in colorectal tumors based on tumor sideness and mutation status, providing evidence for the combination therapy targeting immune checkpoints, growth factors and cytokine signaling, and providing additional support for the individualized treatment of colorectal cancer based on KRAS/TP53 mutation status.

### Validation the clinical significance using bulk sequencing data

**Figure 7:**
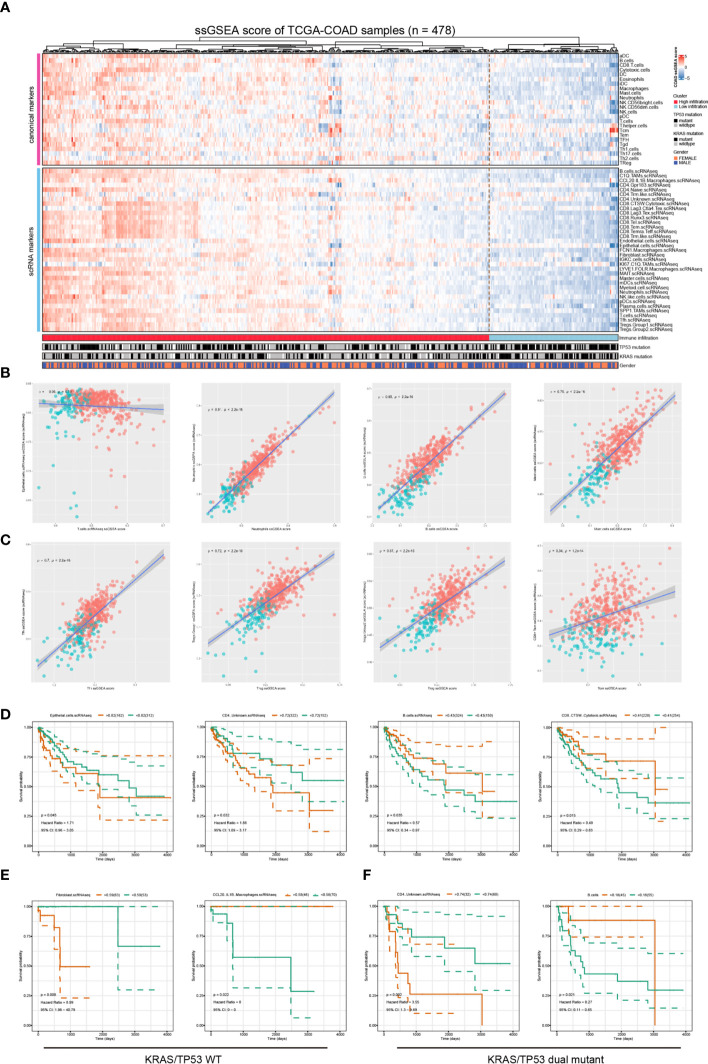
Validation the clinical relevance of the scRNA data using the TCGA-COAD dataset. **(A)** The Heatmap depicted the immune infiltration score in TCGA-COAD cohort (n = 478) based on a group of canonical immune cell markers and cell markers genes identified based on the scRNA-seq analysis. **(B, C)** The correlation between immune infiltration score determined by canonical markers and that identified using scRNA-seq identified marker genes. The spearman correlation rho- and p-value were presented in each figure. **(D)** Survival analyses showed that increased epithelial score and CD4^+^ unknown T cell score were associated with poorer prognosis, and increased B cell score and CD8^+^ CTSW^+^ Cytotoxic T cell score were associated with a better prognosis in the TCGA-COAD cohort. The optimal separation points of the continues immune infiltration indicators were identified using the R survminer algorithms. **(E)** In patients with KRAS/TP53 wildtype colorectal cancer, the fibroblast infiltration score predicted worse prognosis, whereas the CCL20^+^IL1B^+^ macrophages infiltration predicted better prognosis. **(F)** In patients with KRAS/TP53 dual mutant colorectal cancer, the CD4^+^ unknown T cell score indicated poorer prognosis, and the B-cell infiltration score indicated a favorable prognosis.

Finally, we validated our findings using bulk sequencing data from TCGA-COAD cohort (n= 478) using ssGSEA analysis. We quantified the immune cell components in the bulk sequencing data using both canonical ssGSEA markers and scRNA-seq identified marker genes. The heatmap of the immune landscape and corresponding clinical information was presented (**Figure 7A**). The samples were categorized into two categories, the high immune infiltration and low immune infiltration group, using unsupervised clustering (**Figure 7A**). There was no correlation between T-cell and epithelial-cell infiltration score in the patient cohort, suggesting that the ssGSEA makers could efficiently distinguish the major cellular components in colorectal cancers (**Figure 7B**, left). The infiltration score of the same cell type was evaluated using single-cell markers and canonical gene sets, and the results showed a high degree of congruence (**Figures 7B, C** **)**. We further investigated the correlation between the immune infiltration score of these 59 immune cells and the prognosis of colorectal cancers (**Figure 7D**). The results showed that increased epithelial score and CD4^+^ unknown T cell score were associated with a poorer prognosis, while increased B cell score and CD8^+^ CTSW^+^ Cytotoxic T cell score were associated with a better prognosis in the TCGA-COAD cohort (**Figure 7D**). After stratifying patients based on tumor mutations, we observed that fibroblast enrichment predicted a poor prognosis, whereas CCL20^+^ IL1B^+^ macrophage enrichment indicated significantly prolonged survival in KRAS/TP53 wild-type tumors (**Figure 7E**; **Supplementary Figures 6A–D**). Meanwhile, CD4^+^ unknown T cell and B cell infiltration score were optimal prognosis indicators in KRAS/TP53 dual mutant tumors (**Figure 7F**). Collectively, our results highlight the importance of diverse immune cells in the prognosis of colorectal cancer; we also discuss the variation in immune cell score in prognosis based on mutation status. The results provided evidence that stratification of the patients might improve the prognosis prediction and efficiency of immunotherapy.

## Discussion

The main objective of this study was to analyze the effects of KRAS/TP53 mutation status and tumor sideness on the immune microenvironment of colorectal cancer using single-cell sequencing data. One notable aspect of our study was to reveal that the immune microenvironment has undergone a gradual remodeling with an enrichment of immunosuppressive myeloid cells from normal mucosa to tumor regions. Our results revealed the metabolic heterogeneity of tumor-infiltrating immune cells, and highlighted that KRAS/TP53 dual mutation may impair the antitumor immunity *via* reducing the energy metabolism of T cells. These findings strengthened that optimizing energy consumption may be a viable strategy for enhancing the immunotherapeutic efficacy for colorectal tumors (53, 54). Another notable aspect of our study was to elucidate the distinction in cellular interactions in KRAS/TP53 mutant and wildtype tumors. The pro-tumoral cellular interactions, such as HBEGF-CD9 and CD24-SIGLEC10, could be potential therapeutic targets for colorectal cancers bearing KRAS/TP53 mutations.

The use of single-cell sequencing to resolve the characteristics of cancer heterogeneity and immune microenvironment has gained a great deal of attention in recent years. Zhang and colleagues systematically characterized the tumor microenvironment of colorectal cancer patients, revealing the immune characteristics, lineage development, and cell-cell communication of tumor-associated macrophages. This study mainly focused on the tumor infiltrating myeloid cells, and revealed the potential mechanism of immunotherapeutics targeting myeloid cells (anti-CSF1R and anti-CD40 agonism) in mouse models with colorectal cancers (32). Meanwhile, taking the advantages of single-cell multi-omics sequencing, Zhou et al. provided evidence that somatic copy number alternations were prevalent in immune cells, fibroblasts and endothelial cells in both normal tissue and colorectal cancer. The authors highlighted that the overexpressed BGN, RCN3, TAGLN, MYL9 and TPM2 in cancer-associated fibroblast as potential prognostic biomarkers for patients with colorectal cancers (55). More perspectives for resolving the tumor microenvironment have emerged as a result of the use of single-cell technologies. However, the relation between tumor microenvironment and immunometabolism remains unknown. In the present study, we particularly focused on the metabolic differences of immune cells in colorectal cancer across different mutation status. Multiple immune- and metabolic-associated pathways of CD8^+^ and CD4^+^ T cells were dramatically dysregulated in colorectal tumors harboring KRAS/TP53 mutations, impairing their anti-tumor functions. Further analysis showed that KRAS/TP53 dual mutations significantly reduced the major energy metabolic functions in immune cells, especially in T cells. This energy deficiency tends to greatly inhibit the anti-tumor immune function of T cells. Previous studies have confirmed that KRAS-driven cancer cells altered the content and structure of the tumor microenvironment, hence altering nutrient metabolism and oxygen availability in solid tumors, modifying the tumor microenvironment and limiting the proliferation and activity of T cells (56–58). Moreover, lactic acid releasing as a result of tumor glucose metabolism also substantially polarized macrophages towards immunosuppression (59, 60). Additionally, we found that the expression LAG3 is a critical immune checkpoint for CD8^+^ Tex, and these CD8^+^ Tex were categorized into LAG3^+^ CD8^+^ Tex and CTLA4^+^ LAG3^+^ CD8^+^ Tex subgroups. The levels of cytokines released by these CD8^+^ Tex, including GZMA, GZMB, IFN-γ and CXCL13, were dramatically reduced. These findings helped to explain why a subset of colorectal cancer patients did not respond to immunotherapy targeting PD1/PD-L1 or CTLA4 (61, 62), and offered evidence for the use of anti-LAG3 immunotherapy for colorectal cancers (63, 64). In our analysis, the gender group showed a bias phenomenon regarding CD8^+^ Runx3^+^, CD8^+^CTSW^+^ cytotoxic, and CD8^+^ exhaustion. Recently, a series of clinical and experimental studies reported that gender has a significant impact on the effectiveness of antitumor immunotherapy (65–67). In patients with colorectal cancer or melanoma, the androgen receptor (AR) mediated signaling pathways impaired the stem-cell like feature of CD8^+^ T cells, resulting in CD8^+^ T cell exhaustion in male tumor patients, whereas female patients with lower AR expression and androgen levels gained better antitumor immunity (66). Similarly, a subsequent study reported that androgen promoted the exhaustion of CD8^+^ T cell, which accelerated the tumor growth. Blockade of the androgen mediated signaling pathways reshaped the tumor microenvironment, promoted the differentiation of effector T cells, and enhanced the immunotherapeutic efficacy (67). Our findings also implied that female patients may retain stronger CD8^+^T cell derived antitumor immunity. This result, however, has to be examined and validated in a larger group of patients. Taken together, these findings, corroborated previous findings, added additional evidence for the cross-talk between KRAS/TP53 mutations status and the immune microenvironment at the single-cell resolution. In clinical practice, KRAS status has been used to determine whether colorectal cancer patients should receive chemotherapy, bevacizumab, or cetuximab. These new data suggested that targeting tumor metabolism, both through diet or medication, in combination with chemotherapy or immunotherapy might be beneficial in the treatment of colorectal tumors.

Tumor-associated macrophages (TAMs), being the most plastic immune cells in tumors, are functionally and phenotypically diverse in our cohort. We also investigated the function of the myeloid-derived cells from the metabolic perspective. Despite the existence of multiple well-characterized colorectal-cancer-associated macrophage subgroups, including C1QC^+^ TAMs, SPP1^+^ TAMs, and FCN1^+^ TAMs (32, 68, 69), we identified a kind of tumor-resident FOLR2^+^ LYVE1^+^ macrophage subgroup in colorectal cancer. Recently, Rodrigo et al. have revealed a correlation between the density of FOLR2^+^ macrophages and tumor-infiltrating CD8^+^ T lymphocytes in breast cancer. The results showed that increased density of FOLR2^+^ macrophages was associated with favorable survival in breast cancer patients (70). However, the status and function of FOLR2^+^ macrophages in colorectal cancer are poorly characterized. By integrating scRNA-seq data, immunofluorescent staining, and TCGA datasets, we identified that FOLR2^+^LYVE1^+^ macrophages in colorectal cancers, and exhibited steady-state macrophage transcriptional profile and moderate-to-low energy metabolic features. In contrast to breast cancers, the survival data from the Human Protein Atlas and TCGA-COAD cohort showed that FOLR2 mainly indicated an unfavorable prognosis in colorectal cancer, implying its pro-tumor immunological function in colorectal tumors. This finding could be explained by the remarkable plasticity of macrophages in different tumor microenvironments, which requires additional validation of experimental and clinical data. Taken together, these findings provided new insights into the diversity and dynamics of immune cells in colorectal cancer.

In our results, the malignant cells had the highest heterogeneity across individuals, whereas immune cells showed less heterogeneity, which was consistent with earlier bulk-sequencing data revealing a high degree of heterogeneity in colorectal cancer (15, 71). Solofa and colleagues used a bulk high-depth sequencing approach to decode the single nucleotide (SNV) and gene copy number variant (CNV) heterogeneity in colorectal cancer (71). They discovered universal APC and TP53 mutations in all tumors, as well as more highly variable gene copy numbers in a variety of genes. While our findings are consistent with these, we also validated the CNV heterogeneity of colorectal cancers at the single-cell level. We observed that the majority of tumor cells share a common trunk clone, whereas numerous distinct subclones emerge across various samples. This data, in conjunction with the finding from the evolution tree analysis, suggested that colorectal cancers could undergo trans-differentiation from normal epithelial cells, enhancing our understanding of the heterogeneity and molecular mechanisms of colorectal cancers.

There are several limitations of our study. First, the batch effect cannot be completely eliminated by the statistical methods. We realized cellular heterogenicity across different patients, and that integrating the datasets using harmony software would inevitably result in some information loss. Second, the biological characteristics of the tissues utilized for single-cell sequencing may differ from those used for pathological examination, and the results of scRNA-seq should be validated further to determine whether they are representative of the entire tumor, and the functions of plasma cells and B cells also need further investigations. The cell-cell interactions were investigated in our study. Although single-cell assays can provide a more comprehensive landscape than previous reported immunofluorescent staining-based assays (72). Single-cell RNA sequencing and bioinformatic analysis are only preliminary steps in the identification of immune checkpoint pathways. Additional experiments are necessary to validate the functions of these immune checkpoints (73–75). For instance, flow cytometry, immunofluorescent staining, and *in situ* hybridization analysis are important methods to verify the expression patterns of the immune checkpoints (73, 76). The function of immune checkpoint ligand-receptor pairs should also be interrogated using a co-culture system. For example, Mathewson et al. interrogated the CD161-CLEC2D pathway using a co-culture system with genomic-edited T cells and patient-derived glioma cells to identify CD161 as an inhibitory receptor for tumor-specific T cells (73). Targeted immune checkpoint blockade is also an important method for functional validation of immune checkpoint pairs (76). Finally, flow cytometry analysis combined with high-throughput screening with cell microarrays individually expressing potential membrane protein targets is a key method to confirm the binding partners of immune checkpoint molecules (75). Mechanically, it still remains unclear how the KRAS/TP53 mutant cancer cells affect the function of tumor infiltrating T cells and macrophages. Therefore, further experiments are required.

## Conclusions

In conclusion, this comprehensive analysis contributed to the strengthening of the sample representation and the identification of novel immune regulatory mechanisms in colorectal cancers. Notably, our data significantly advanced our understanding of the impact of tumor-sideness and mutations on the microenvironment of tumors, particularly immune cell metabolism. This stratification analysis improved our understanding of the heterogeneity and immune metabolism in colorectal cancer, thus providing additional therapeutic targets.

## Data availability statement

The original contributions presented in the study are included in the article/Supplementary Material. Further inquiries can be directed to the corresponding authors.

## Ethics statement

The studies involving human participants were reviewed and approved by The ethics committee of Ruijin Hospital affiliated to Shanghai Jiao Tong University School of Medicine. The patients/participants provided their written informed consent to participate in this study.

## Author contributions

Conceptualization, XL and ZMW; methodology, XL, ZW and XX; software, XL and ZZW; validation, ZMW, WH and ZYW, formal analysis, ZZW and XL; investigation, XX; resources, XX; data curation, XX; writing—original draft preparation, XL, XX and ZZW; writing—review and editing, ZMW, XD and ZYW; visualization, ZZW and XX; supervision, ZMW and WH; project administration, ZMW and WH; funding acquisition, ZMW and XL. All authors have read and agreed to the published version of the manuscript.

## Funding

This work was founded by the National Natural Science Foundation of China (No.81771949, receiptant: ZMW), The Ruijin Hospital fund for Young Scholars (No. KY2022001, receiptant: XL), Shanghai Key Specialty Construction Project (NO. ZK2019A02, receiptant: ZMW), and the Shanghai Jiao Tong University Translational Medicine Research Fund (No. ZH2018ZDA04, receiptant: ZMW).

## Acknowledgments

We thank Dr. Jianming Zeng (University of Macau), and all the members of his bioinformatics team, bio-trainee, for generously sharing their experience and codes. We thank all participants in this study.

## Conflict of interest

The authors declare that the research was conducted in the absence of any commercial or financial relationships that could be construed as a potential conflict of interest.

## Publisher’s note

All claims expressed in this article are solely those of the authors and do not necessarily represent those of their affiliated organizations, or those of the publisher, the editors and the reviewers. Any product that may be evaluated in this article, or claim that may be made by its manufacturer, is not guaranteed or endorsed by the publisher.

## Supplementary material

Supplementary Table 1 The clinical information of the patients included in the analysis.

Supplementary Table 2 The key genes enriched in each stage of CD4^+^ and CD8^+^ T cell pseudotime analysis.

Supplementary Table 3 The global and functional cell-cell communication intensity in colorectal cancers.

Supplementary Figure 1
**(A)** The schematic workflow for literature search. **(B)** The proportion of major immune and non-immune cell types across different samples. The bubble plot presented the expression of typical cell marks across the cellular clusters in colorectal cancers. **(C)** The heatmap represented the proportion of 9 cell types across different samples. **(D)** The barplot represented cell proportion regarding each phenotype including tumor location, KRAS/TP53 mutation status, gender and patient cohort. The tumor location indicated the sideness of the colorectal cancer (**left-sided** or **right-sided**). The tumors were categorized in to 4 groups, including KRAS (tumors only bear KRAS mutations), DUAL (tumors bear KRAS and TP53 dual mutations), TP53 (tumors only bear TP53 mutations) and WT (neither KRAS nor TP53 are mutated). The patient cohort information was provided to identify the origins of the patients.

Supplementary Figure 2 Identification of normal and malignant epithelial cells in tumor adjacent and tumor tissue. **(A, B)** The UMAP plots presented the cluster feature and sample identity of all epithelial cells in normal mucosa, tumor adjacent and tumor tissue. **(C)** The heatmap depicted the tissue origin and unsupervised clustering groups of all epithelial cells from normal mucosa, tumor adjacent and tumor tissue according to the CNV analysis. Cluster 4 and 7 were considered as normal epithelial cells.

Supplementary Figure 3 Identify T-cell subtypes in normal mucosa, tumor adjacent and tumor tissues. **(A)** Heatmap depicted the top differentially expressed marker genes across they T cell types. For each group, a maximum of 500 cells were randomly selected to draw the heatmap. **(B)** The heatmap presented the proportion of 16 T-cell types across different samples. **(C)** The differentially expressed genes in CD8+ CTSW+ cytotoxic, CD8+ Runx3+, and CD8+ exhausted T cells between the male and female patients. **(D)** The average expression levels of immune checkpoint molecules across the 16 subtypes of T cells according to tumor sideness. The bar indicated the row-scaled expression of immune checkpoint molecules.

Supplementary Figure 4 Tumor-infiltrating T cells exhibit molecular heterogeneity according to tumor sideness and KRAS/TP53 mutation status. (**A, B**) The UMAP plots demonstrated the distribution of 16 kind of T-cell subtypes according to tissue origin **(A)** and tumor sideness **(B)**. **(C)** The GSEA analysis of DGEs demonstrated the differently enriched pathways of T cells in left- and right-sided colorectal cancers. **(D)** The UMAP plots demonstrated the distribution tumor-infiltrating T-cell subtypes according to KRAS/TP53 mutation status. **(E)** he GSEA analysis of DGEs demonstrated the differently enriched pathways of tumor-infiltrating T cells according to KRAS/TP53 mutation status.

Supplementary Figure 5 Identify the myeloid-cell subtypes in normal mucosa, tumor adjacent and tumor tissues. **(A)** Heatmap depicted the top differentially expressed marker genes across they myeloid cell types. For each group, a maximum of 500 cells were randomly selected to draw the heatmap. **(B)** The heatmap presented the proportion of 10 myeloid-cell types across different samples. **(C)** The t-SNE plots demonstrated the distribution of 10 myeloid-cell subtypes according to tumor sideness.

Supplementary Figure 6 Survival analyses identified the prognostic value of immune-infiltration score of 59 immune cells according to the KRAS/TP53 mutation status. **(A–D)** represented the statistically significant immune-infiltration predictors in **(A)** KRAS/TP53 wildtype, **(B)** TP53 mutant, **(C)** KRAS mutant, and **(D)** KRAS/TP53 dual mutant colorectal cancers.

## Abbreviations

PD-1, programmed death 1; CTLA-4, cytotoxic T-lymphocyte-associated protein 4; TME, tumor immune microenvironment; MDSC, myeloid-derived suppressor cells; TCGA, the cancer genome atlas; TPM, Transcripts per Million; t-SNE, t-distributed stochastic neighbor embedding; UMAP, uniform manifold approximation and projection; PCA, principal component analysis; GSEA, gene set variant analysis; CNV, copy number variation; TIL, tumor infiltrating lymphocyte.
