# Supplementary figures and images for: Integrated single-cell RNA-seq analysis identifies immune heterogeneity associated with KRAS/TP53 mutation status and tumor-sideness in colorectal cancers
Source: Front Immunol. 2022 Sep 12;13:961350. doi: 10.3389/fimmu.2022.961350 (PMC9510840; doi:10.3389/fimmu.2022.961350)

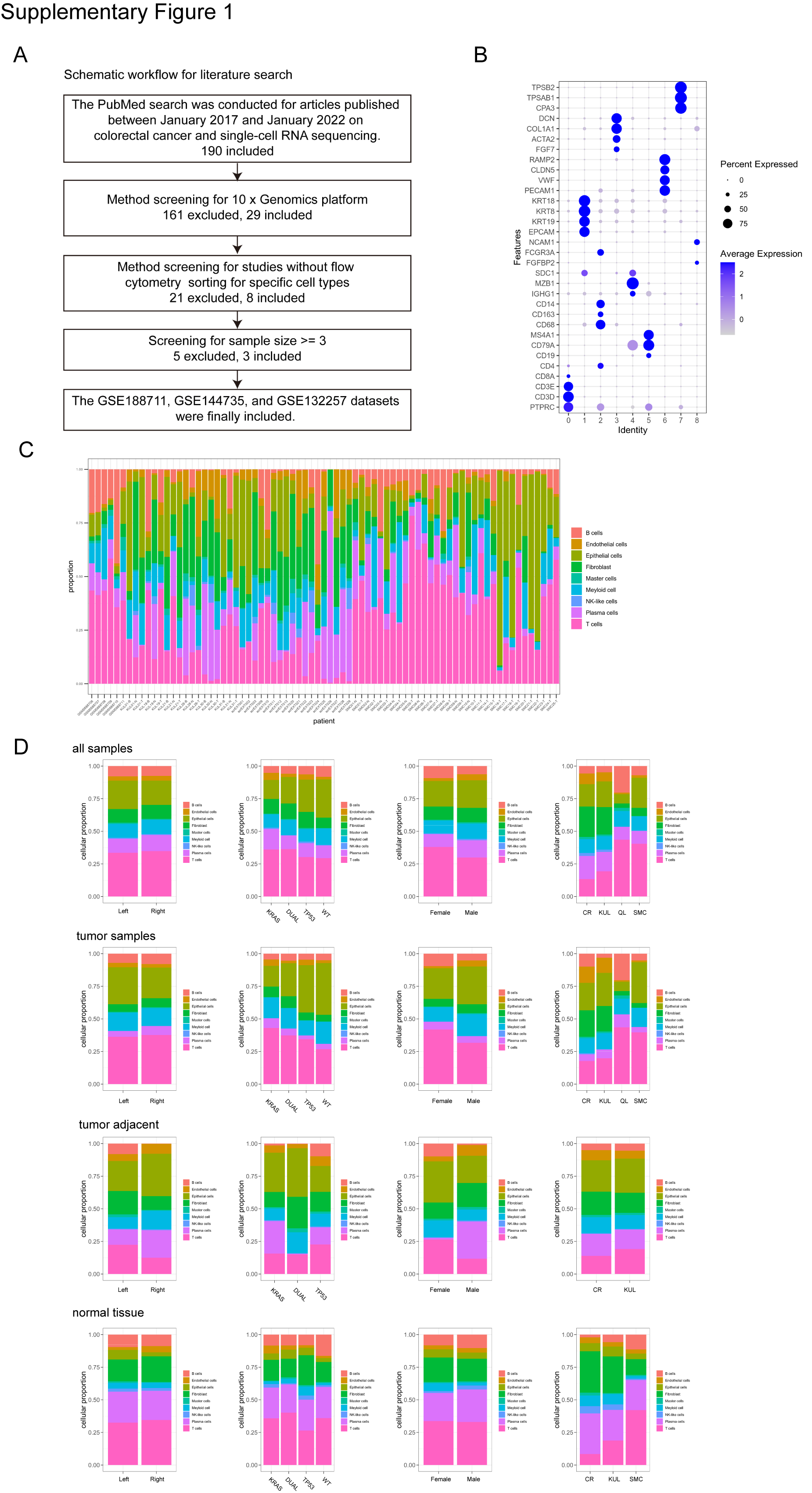

Supplement: Supplementary Figure 1 — (A) The schematic workflow for literature search. (B) The proportion of major immune and non-immune cell types across different samples. The bubble plot presented the expression of typical cell marks across the cellular clusters in colorectal cancers. (C) The heatmap represented the proportion of 9 cell types across different samples. (D) The barplot represented cell proportion regarding each phenotype including tumor location, KRAS/TP53 mutation status, gender and patient cohort. The tumor location indicated the sideness of the colorectal cancer (left-sided or right-sided). The tumors were categorized in to 4 groups, including KRAS (tumors only bear KRAS mutations), DUAL (tumors bear KRAS and TP53 dual mutations), TP53 (tumors only bear TP53 mutations) and WT (neither KRAS nor TP53 are mutated). The patient cohort information was provided to identify the origins of the patients. [file Image_1.tif]

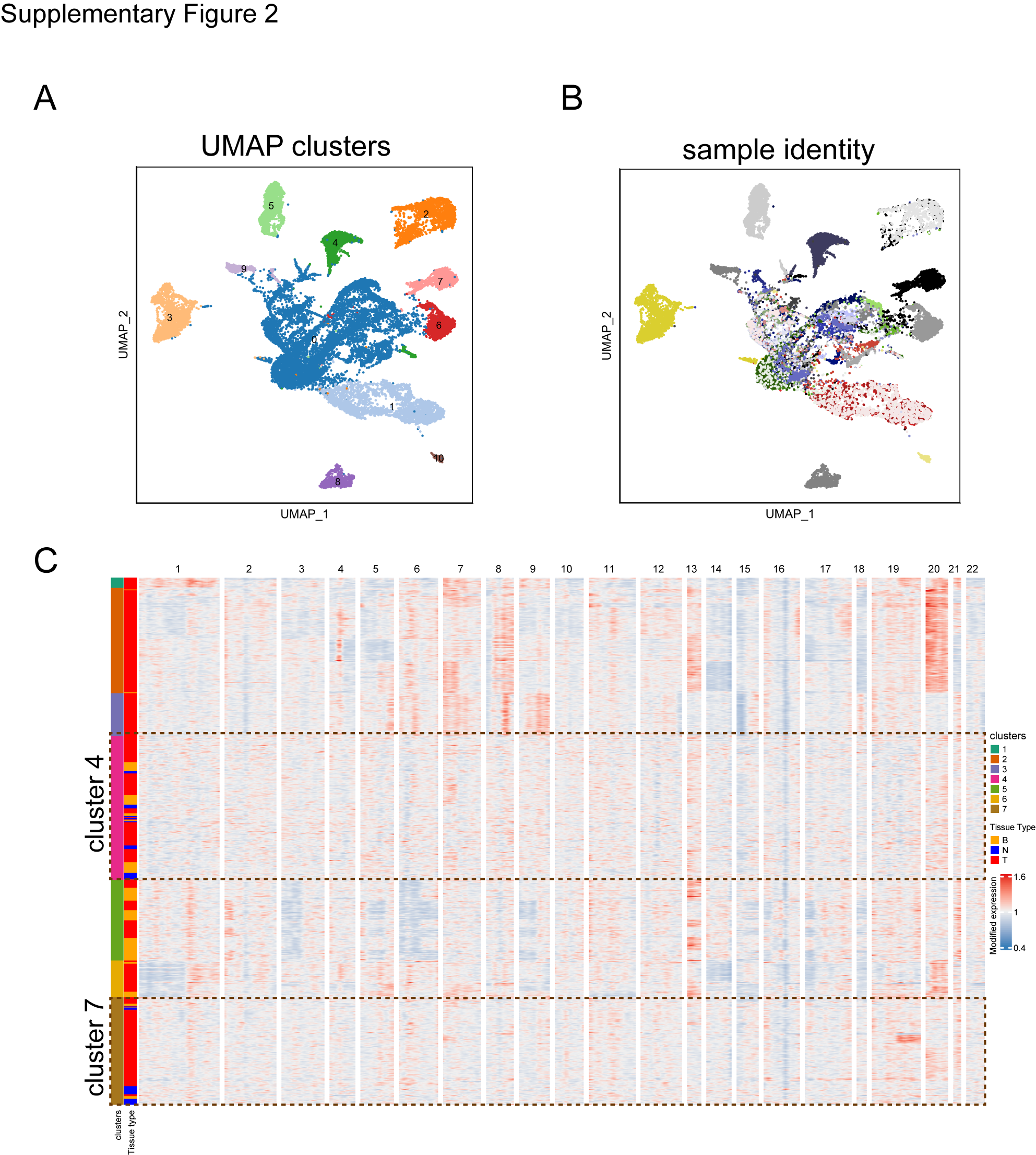

Supplement: Supplementary Figure 2 — Identification of normal and malignant epithelial cells in tumor adjacent and tumor tissue. (A, B) The UMAP plots presented the cluster feature and sample identity of all epithelial cells in normal mucosa, tumor adjacent and tumor tissue. (C) The heatmap depicted the tissue origin and unsupervised clustering groups of all epithelial cells from normal mucosa, tumor adjacent and tumor tissue according to the CNV analysis. Cluster 4 and 7 were considered as normal epithelial cells. [file Image_2.tif]

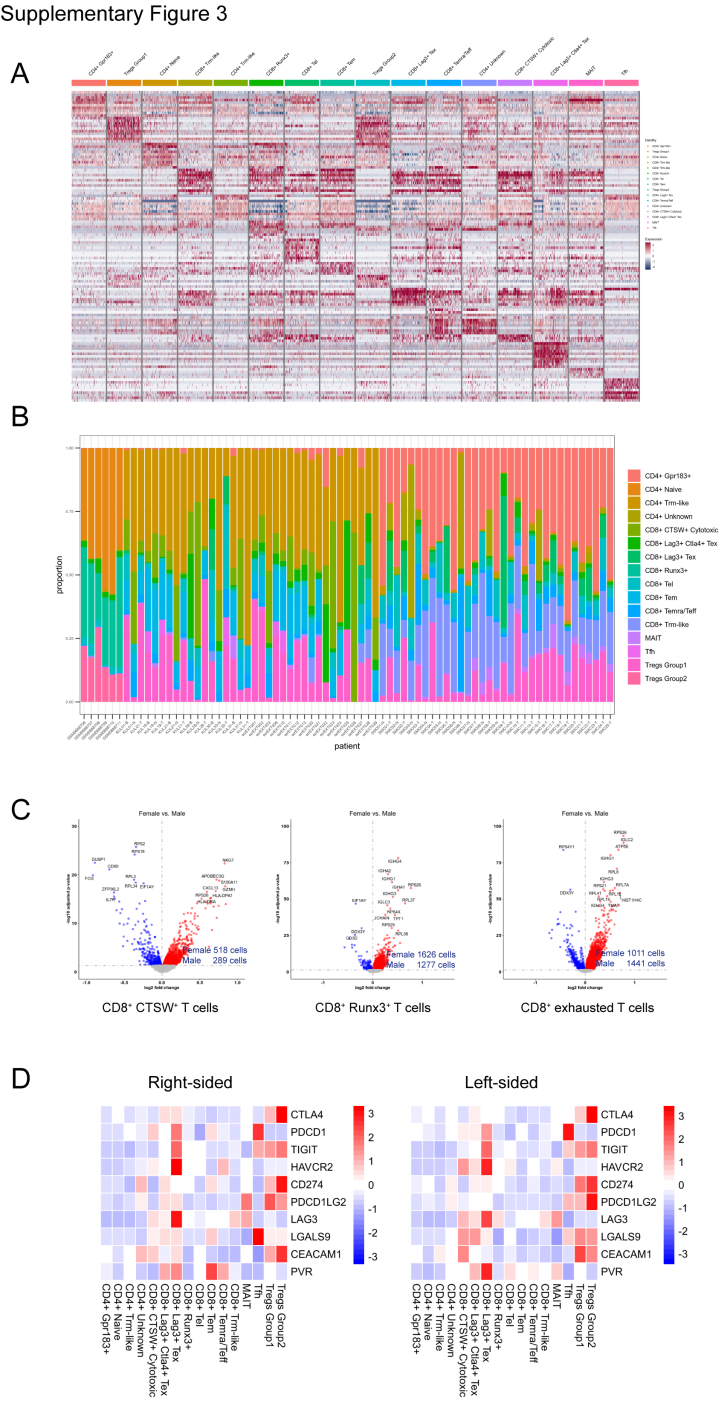

Supplement: Supplementary Figure 3 — Identify T-cell subtypes in normal mucosa, tumor adjacent and tumor tissues. (A) Heatmap depicted the top differentially expressed marker genes across they T cell types. For each group, a maximum of 500 cells were randomly selected to draw the heatmap. (B) The heatmap presented the proportion of 16 T-cell types across different samples. (C) The differentially expressed genes in CD8+ CTSW+ cytotoxic, CD8+ Runx3+, and CD8+ exhausted T cells between the male and female patients. (D) The average expression levels of immune checkpoint molecules across the 16 subtypes of T cells according to tumor sideness. The bar indicated the row-scaled expression of immune checkpoint molecules. [file Image_3.tif]

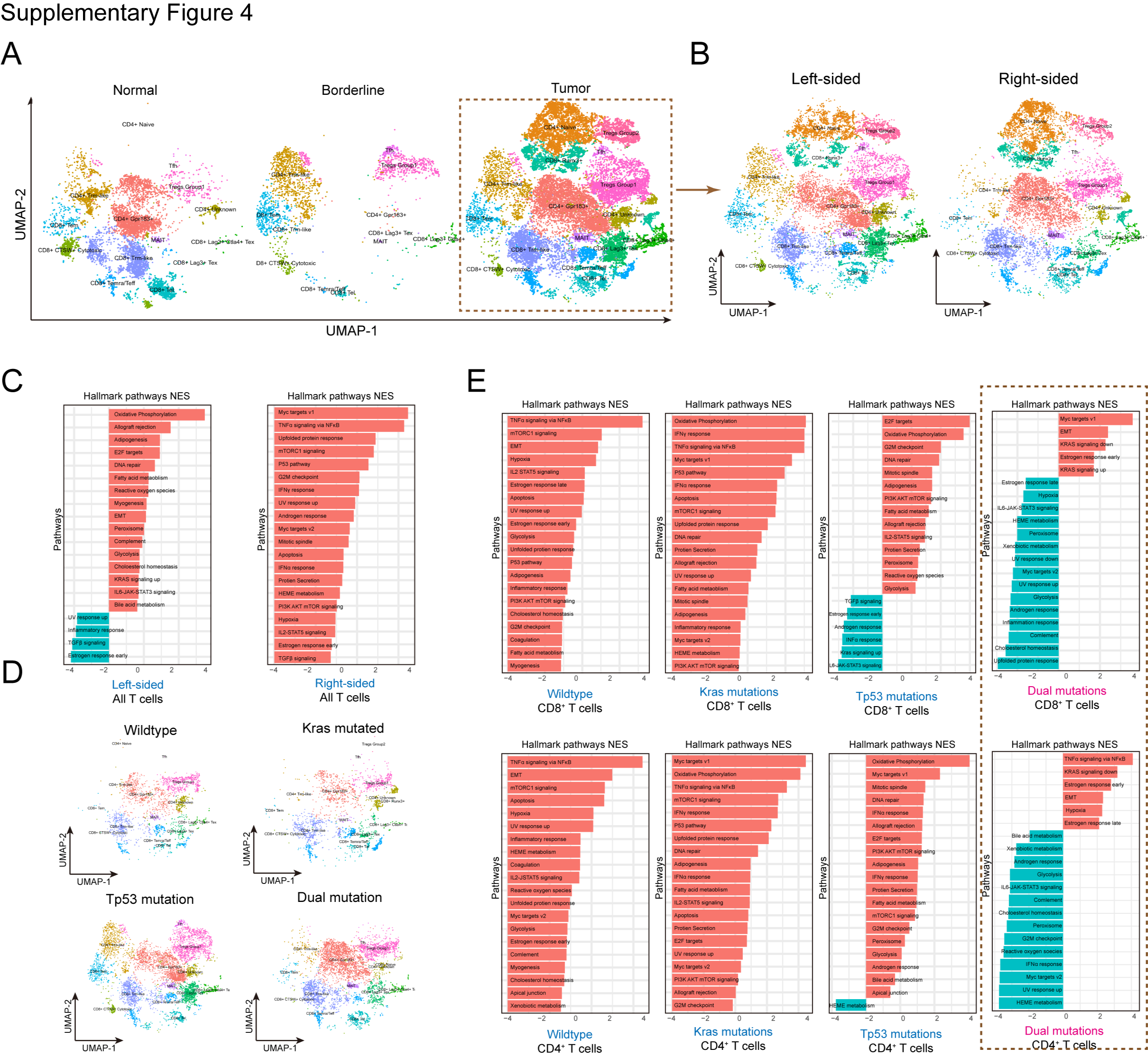

Supplement: Supplementary Figure 4 — Tumor-infiltrating T cells exhibit molecular heterogeneity according to tumor sideness and KRAS/TP53 mutation status. (A, B) The UMAP plots demonstrated the distribution of 16 kind of T-cell subtypes according to tissue origin (A) and tumor sideness (B). (C) The GSEA analysis of DGEs demonstrated the differently enriched pathways of T cells in left- and right-sided colorectal cancers. (D) The UMAP plots demonstrated the distribution tumor-infiltrating T-cell subtypes according to KRAS/TP53 mutation status. (E) he GSEA analysis of DGEs demonstrated the differently enriched pathways of tumor-infiltrating T cells according to KRAS/TP53 mutation status. [file Image_4.tif]

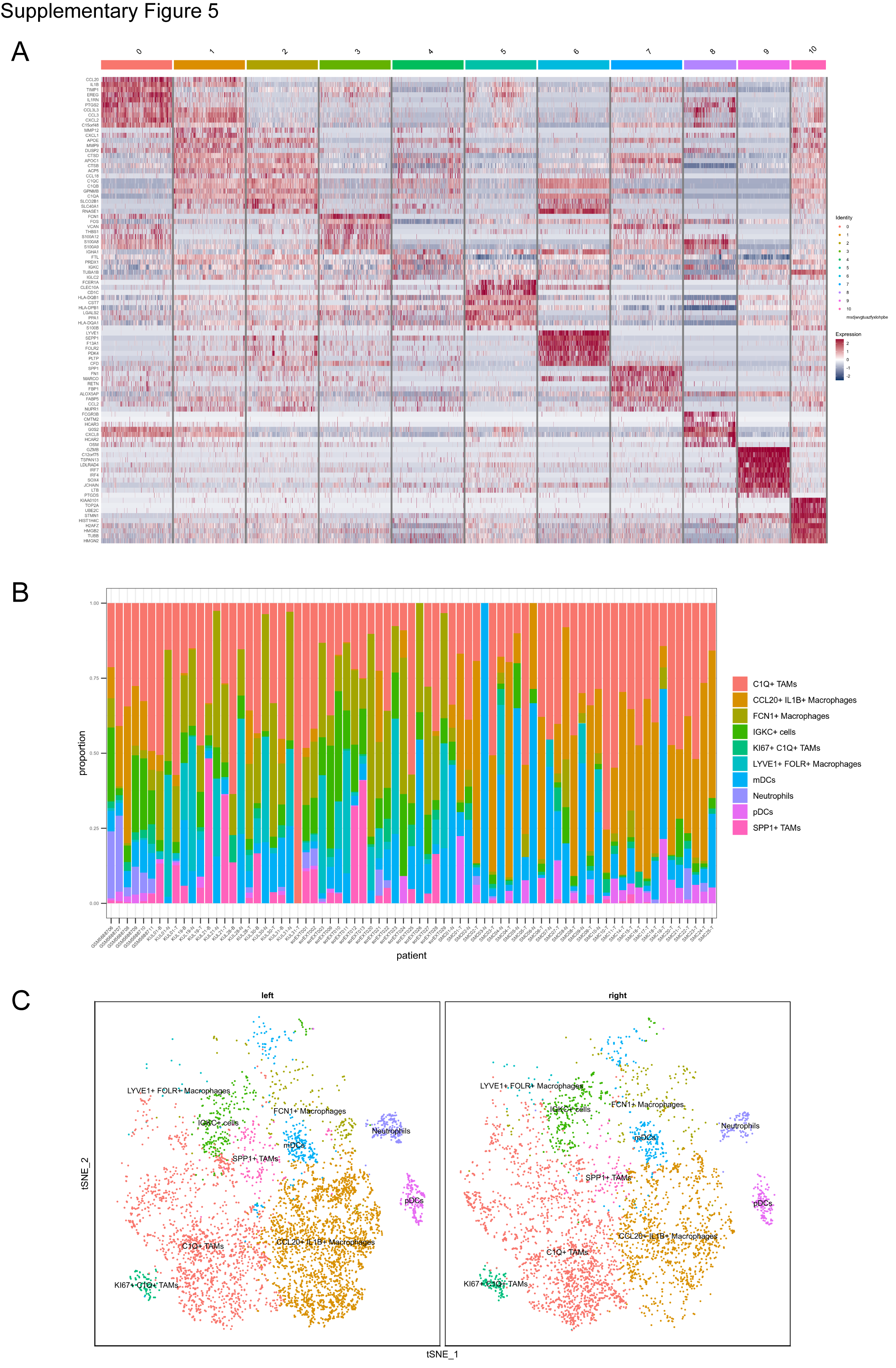

Supplement: Supplementary Figure 5 — Identify the myeloid-cell subtypes in normal mucosa, tumor adjacent and tumor tissues. (A) Heatmap depicted the top differentially expressed marker genes across they myeloid cell types. For each group, a maximum of 500 cells were randomly selected to draw the heatmap. (B) The heatmap presented the proportion of 10 myeloid-cell types across different samples. (C) The t-SNE plots demonstrated the distribution of 10 myeloid-cell subtypes according to tumor sideness. [file Image_5.tif]

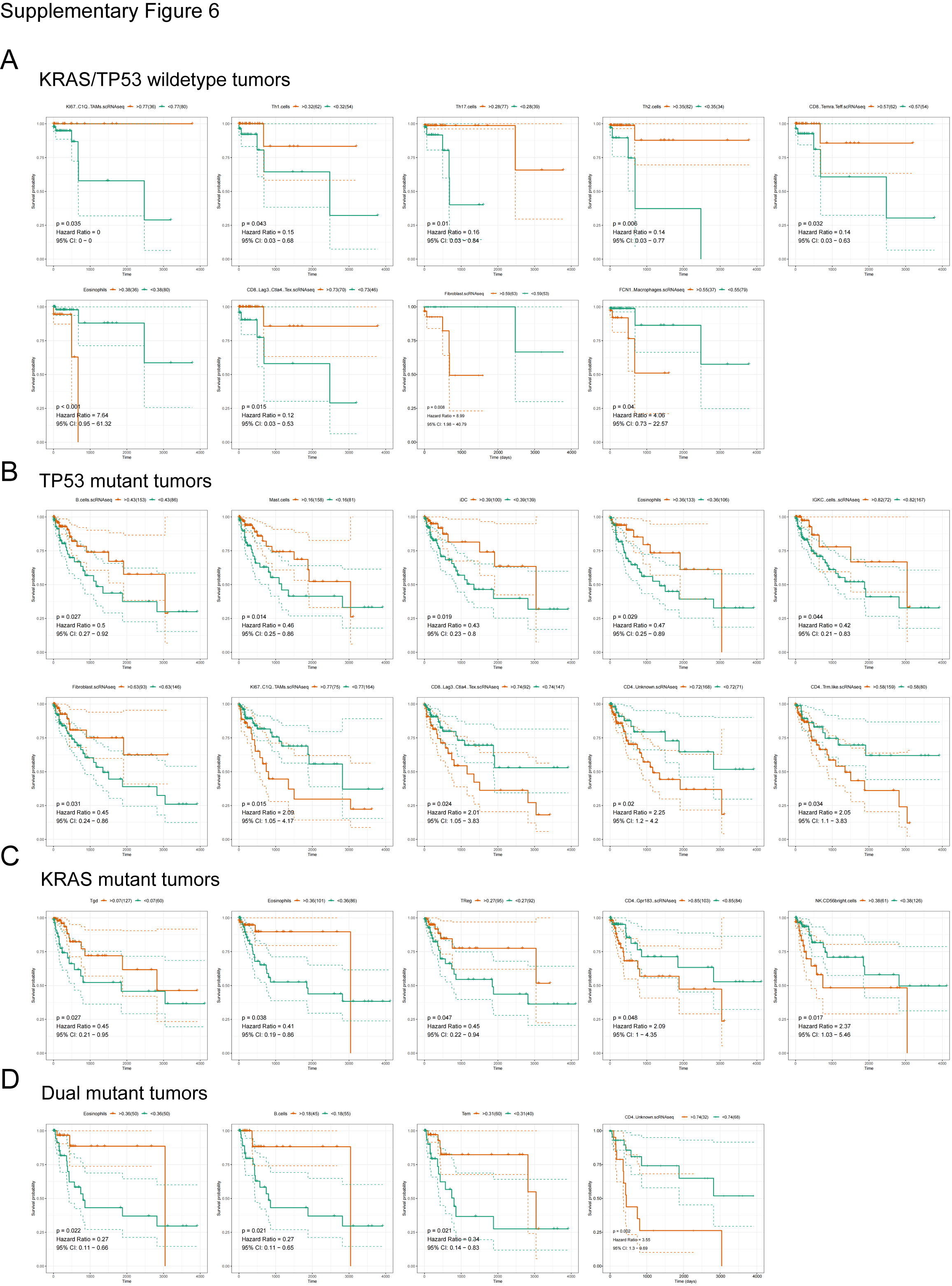

Supplement: Supplementary Figure 6 — Survival analyses identified the prognostic value of immune-infiltration score of 59 immune cells according to the KRAS/TP53 mutation status. (A–D) represented the statistically significant immune-infiltration predictors in (A) KRAS/TP53 wildtype, (B) TP53 mutant, (C) KRAS mutant, and (D) KRAS/TP53 dual mutant colorectal cancers. [file Image_6.tif]
